# A systematic review of geographical variation in access to chemotherapy

**DOI:** 10.1186/s12885-015-2026-y

**Published:** 2015-12-31

**Authors:** Charlotte Chamberlain, Amanda Owen-Smith, Jenny Donovan, William Hollingworth

**Affiliations:** School of Social and Community Medicine, University of Bristol, 39 Whatley Rd, Bristol, BS8 2PS UK

**Keywords:** Cancer, Drugs, Chemotherapy, Variation, Geography, Health inequalities, Systematic review

## Abstract

**Background:**

Rising cancer incidence, the cost of cancer pharmaceuticals and the introduction of the Cancer Drugs Fund in England, but not other United Kingdom(UK) countries means evidence of ‘postcode prescribing’ in cancer is important. There have been no systematic reviews considering access to cancer drugs by geographical characteristics in the UK.

**Methods:**

Studies describing receipt of cancer drugs, according to healthcare boundaries (e.g. cancer network [UK]) were identified through a systematic search of electronic databases and grey literature. Due to study heterogeneity a meta-analysis was not possible and a narrative synthesis was performed.

**Results:**

8,780 unique studies were identified and twenty-six included following a systematic search last updated in 2015. The majority of papers demonstrated substantial variability in the likelihood of receiving chemotherapy between hospitals, health authorities, cancer networks and UK countries (England and Wales). After case-mix adjustment, there was up to a 4–5 fold difference in chemotherapy utilisation between the highest and lowest prescribing cancer networks. There was no strong evidence that rurality or distance travelled were associated with the likelihood of receiving chemotherapy and conflicting evidence for an effect of travel time.

**Conclusions:**

Considerable variation in chemotherapy prescribing between healthcare boundaries has been identified. The absence of associations with natural geographical characteristics (e.g. rurality) and receipt of chemotherapy suggests that local treatment habits, capacity and policy are more influential.

**Electronic supplementary material:**

The online version of this article (doi:10.1186/s12885-015-2026-y) contains supplementary material, which is available to authorized users.

## Background

Cancer is the leading cause of mortality in the United Kingdom (UK) [1]. Cancer incidence is rising and so too is the proliferation of high-cost, life-extending cancer drugs. There are potential restrictions on access to cancer drugs in the UK National Health Service (NHS) at a number of levels: national policy; regional and local commissioner and provider activity; clinician prescribing preferences, and individual patient care seeking behaviour [2–4]. The UK was ranked 12th of 14 European countries for the prescribing of cancer pharmaceuticals launched in the last 5 years [5]. Within the UK, there has been considerable attention on regional variation in prescribing [6, 7] and this was a major factor in the establishment of the National Institute for Health and Care Excellence (NICE) in 1999; an attempt to ameliorate the so called, ‘postcode lottery of prescribing’. The recent restructuring of the NHS, with a move to Strategic Clinical Networks, (SCNs) instead of Cancer Networks; the continued trend for centralisation of cancer services to drive quality and efficiency improvements, and divergent cancer drugs funding policy, with the establishment of the Cancer Drugs Fund in England, but not in Wales, Scotland or Northern Ireland, are all important changes that may impact on equity of cancer pharmaceutical prescribing by geographical region.

‘Access’ to cancer drugs, encompasses the quality, equitability, acceptability and availability of chemotherapy for those in need [8]. The term chemotherapy is frequently used in the literature to represent anti-cancer drugs, although chemotherapy can represent any-drug therapy or specific anti-cancer therapies that exclude hormonal treatments or radiopharmaceuticals for instance. For the purpose of this paper chemotherapy is used interchangeably with anti-cancer drug therapy to capture all relevant papers. It is challenging to measure access and therefore rates of utilisation alone are frequently used as a proxy. Utilisation may appear appropriate for the size of population under study, but instead represent health demand, rather than clinically assessed ‘health need’ and in some circumstances, mask inequitable use of services [9]. Furthermore, variation may result from explicit resource allocation decisions, such as the decision by Wales not to have a Cancer Drugs Fund, prioritising spend earlier in the cancer pathway. However, poor uptake of cancer drugs has been associated with reduced life expectancy in correlational studies [10] and therefore, variation in use or access to these drugs where it is unwarranted according to clinical need may represent a threat to health. Variation may be considered inequitable and contrary to the NHS constitution [11] if it is the result of opaque healthcare boundary differences in provision or ‘natural’ geographical barriers (e.g. distance).

Three systematic [12–14] and seven literature reviews [3, 4, 15–19] have considered diffusion of pharmaceuticals and innovations in developed and developing nations alongside distribution and uptake of other cancer treatment modalities. However, none of these reviews are systematic accounts of barriers to access to cancer pharmaceuticals. The international literature includes surveys of patient perceptions of the role of rurality or distance to treatment in their chemotherapy decision-making and both found evidence that the distance to treatment has an influence on uptake or compliance with treatment options for their cancer [20, 21]. Widely cited, grey literature publications in the UK, comparing chemotherapy utilisation by healthcare geographical area: England vs Wales vs Scotland; Strategic Health Authority (SHA) or Primary Care Trust level, have found large variation [22, 23]. Exploring and quantifying this variation and the reasons for variation in chemotherapy prescribing by geographical area is therefore important for quality, equitable care for NHS patients.

We aimed to systematically identify published studies considering geographical barriers to *use of cancer pharmaceuticals in the UK NHS*.

## Methods

### Search Strategy

The review methodology was performed in accordance with the Centre for Reviews and Dissemination (National Institute for Health Research [NIHR]) guidance on systematic reviews [24, 25] and reported according to the PRISMA statement, checklist (Additional file 1) and flow-diagram [26]. A systematic literature search was carried out using electronic databases, electronic citation tracking (ISI Web of Knowledge citation indexes), hand-searching of references identified in eligible studies, and grey-literature searching. The search strategy was tailored to each electronic database to account for differing wildcards and system features. Search terms included key words, synonyms and MeSH terms for cancer drugs OR access OR inequality. The search strategy was written by the first author and refined by a medical librarian (CB) and an experienced systematic reviewer (MB). Scoping took place between September and December 2012, with a formal search run by the experienced systematic reviewer (MB) in March 2013. An update of the electronic database search was conducted in July 2015 by the first author (CC). A list of the nine interrogated electronic databases, including MEDLINE and EMBASE is available in the Additional file 2 along with the search strategy. Informal approaches are also described in the Additional file 2 and included Google and specific health and health policy websites (last updated in July 2013).

The search strategy was kept deliberately broad to include all potential barriers to chemotherapy utilisation, including policy and system barriers; environmental context obstacles (including geographical barriers), and challenges resulting from variation in individual patient characteristics (such as age or gender) affecting professional prescribing and appropriateness of services.

### Study Eligibility

Papers were classified according to three themes: ‘policy/systems’, ‘geographical’ and ‘individual patient characteristics’, and where eligible for more than one theme, were included in all suitable themes. Eligible studies which *did not have geographical exposure variables were excluded from this report*, for future study under other theme headings. The primary outcome measure was receipt of chemotherapy, defined by prescribing data.The exposure was geographical healthcare boundary (e.g. cancer network, strategic health authority, acute hospital trust), or other measured geographical characteristic, such as population density (rurality), distance to treatment centre, or travel-time to treatment centre. Inclusion criteria includeddescription of cancer pharmaceutical prescribing in adults (>18 years) in the UK NHS. All cancer drug prescribing, including reports of sub-optimal or delayed prescribing were included. Geographical chracteristics of natural geographical boundaries (testing the influence of rurality, time or distance to treatment) or healthcare geographical boundaries (including healthcare designated areas arranged by policy or organisation, such as acute trust or cancer network) and their influence on chemotherapy receipt were all eligible for inclusion. There were no time or language limitations to the eligibility criteria. Exclusion criteria included papers which focused on all pharmaceuticals and not primarily cancer pharmaceuticals, conference proceedings, quantitative papers with <30 participants or commentary pieces. Study quality was considered, but studies were not excluded on grounds of quality alone.

### Study Selection

Title and abstract screening was performed by CC using EndNote software, with independent double-screening (JB) of a sample of abstracts to assess reliability (5 % of CC excluded abstracts and all CC included abstracts). In the event of uncertainty, studies were included for full-text review. Disagreements were to be resolved with discussion and consensus between JB, CC and WH.

### Data Extraction

Pre-defined data items, as per the STrengthening the Reporting of OBservational studies in Epidemiology (STROBE) checklist headings (e.g. study design, outcome and exposure variables, methods, analysis approach and results amongst others) and study description (author, year, title, journal), whether the manuscript was peer-reviewed, and whether it met the eligibility criteria and if not, why not (reason for exclusion) were extracted from each full-text study in Microsoft Access (CC). Principle summary measures included Odds Ratios (odds of receipt of chemotherapy by the geographical exposure) as well as descriptive statistics with proportions and percentages and appropriate statistical tests (e.g. t-test).

### Analysis

Reporting clarity was evaluated with the STROBE observational checklist and methodological quality with the NICE adapted Graphical Appraisal Tool for Epidemiological studies (GATE) [27]. Narrative synthesis was performed by the first reviewer (CC) and verified by the senior author (WH). The objectives of the systematic review were peer-reviewed as part of the NIHR Doctoral Fellowship award. A protocol was not publicly listed.

## Results

**Fig. 1:**
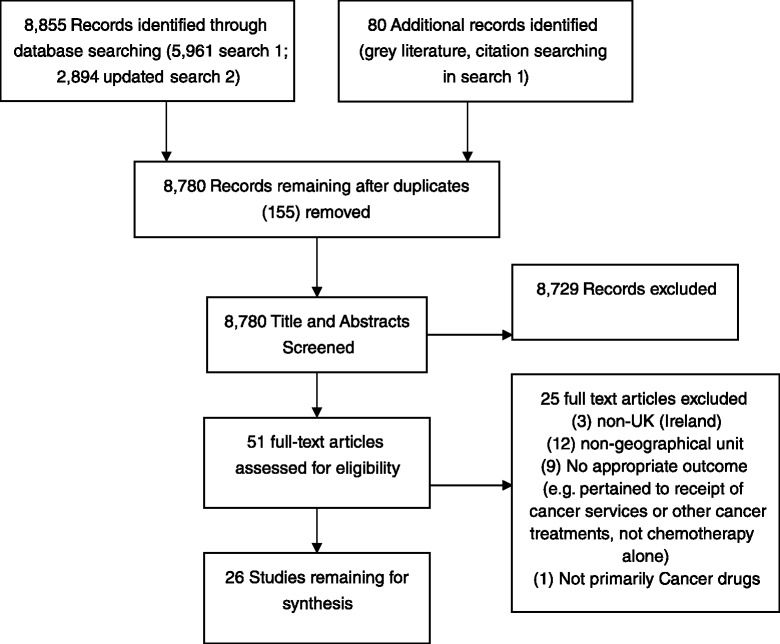
Prisma diagram: access to cancer drugs in the NHS

5,987 unique titles and abstracts (5,961 identified from electronic bibliographic databases and 80 from other sources) were screened, after removal of duplicates (54) in March 2013. An additional 2,894 studies were identified from a repeat of the systematic search strategy of the included electronic databases (July 2015, 2,793 after removal of duplicates [101]). Of 344 double-screened included and excluded abstracts, one initially excluded paper was included in the final analysis. Initial exclusion referred the paper to a different theme and was an error based on mistaking the use of geographical exposure variables, as well as individual characteristics, as part of the study design. Twenty six papers, following the update, were included in the analysis: 16 peer-reviewed and 10 from the grey literature (Fig. 1). Nine grey literature studies were annual National Lung Cancer Audit (NLCA) reports [28]. For simplicity, only one NLCA report is presented in the tables and referenced in the report. All earlier reports follow the same template and are available online. Six included studies referred to multiple cancer types [6, 29–33] 15 to lung cancer only [34–39] four to colorectal cancer [40–43], and one in prostate cancer [44]. Table 1 presents study characteristics and Tables 2–7 present the key findings. Additional file 3: Table S1 describes the reporting quality and Additional file 4: Table S2–S8 the methodological quality of the included studies.

**Table 1:** Study characteristics

Author, Year	Setting	Data sources	Participants	Exposure	Outcome	Statistical methods
Cohort studies						
Beckett ‘12 [34]	England, Wales, Scotland, Northern Ireland and Jersey	National Lung Cancer Audit (NLCA) data ‘09	32,068 lung cancer participants diagnosed histologically or clinically and excluding cases diagnosed at post-mortem. All cancer units included.	Cancer network	Odds of receipt of chemotherapy in Small Cell Lung Cancer (SCLC) within one audit year, by cancer network.	Logistic regression adjusted for age, sex, performance status, stage and deprivation. IMD deprivation index.
Campbell, ‘02 [29]	Grampian and Highland, Scotland	Scottish Cancer Registry ’95 to‘96 and case notes from hospitals	1,314 colorectal and lung cancer participants, excluding cases diagnosed at post-mortem. All cancer units included. Whether participants were diagnosed histologically and clinically:not stated.	Distance to the nearest cancer centre	Odds of receipt of chemotherapy within one year of diagnosis by distance travelled.	Logistic regression adjusted for age, sex, deprivation, cancer site and Dukes stage and histology (lung: SCLC, NSCLC) and ISS stage, health board of residence, and mode of presentation. Carstairs deprivation index.
Cartman, ‘02 [35]	The 17 districts in Yorkshire and South Humber, England	Northern and Yorkshire Cancer Registry (NYCRIS) ‘86 to ‘94	22,654 lung cancer participants diagnosed histologically or clinically and excluding cases diagnosed at post-mortem. All cancer units included.	Health Authority District of residence	Range, numbers and percent of eligible participants receiving chemotherapy by health authority.	District variation measures presented as a range in numbers and percents.
Crawford, ‘12 [40]	The 17 districts in Yorkshire and South Humber, England	NYCRIS ’94 to ‘02	18,221 Colorectal cancer participants diagnosed histologically or clinically. Not stated whether cases diagnosed at post-mortem were excluded. All cancer units included.	Car travel time to healthcare provider	Odds of receipt of chemotherapy within 6 months of diagnosis by travel time.	Logistic Regression adjusted for age, sex and tumour stage. Analysis stratified by deprivation and travel time with a test for interaction. IMD deprivation score.
Crawford, ‘09 [36]	The 17 districts in Yorkshire and South Humber, England	NYCRIS ’94 to ’02	34,923 Lung Cancer participants diagnosed histologically or clinically and excluding cases diagnosed at post-mortem. All cancer units included.	Car travel time	Odds of receipt of chemotherapy within 6 months of diagnosis by travel time.	Logistic regression adjusted for age and sex. Analysis stratified by deprivation and travel time. There was no adjustment for disease stage. IMD deprivation score.
Jack, ‘03 [37]	South East England	Thames Cancer Registry: ’95 to ‘99	32,818 participants with lung cancer confirmed histologically or clinically and excluding cases diagnosed at post-mortem. All cancer units included.	Health authority of residence	Ranges and medians reflecting variation in receipt of chemotherapy by health authority. The odds of receiving chemotherapy by health authority calculated.	Health authority variation presented as medians and ranges. Multi-level logistic regression (participants nested in hospitals or health authorities) adjusted for age, sex, histology, deprivation, lung cancer incidence, whether first hospital attended was a radiotherapy centre, hospital, tumour stage. Townsend deprivation score.
Jones, ‘08 [31]	The 17 districts in Yorkshire and South Humber, England	NYCRIS ’94 to ‘02	117,097 Lung, breast, colon, rectum, ovary and prostate cancer participants, excluding cases diagnosed at post-mortem. Whether both histologically and clinically diagnosed participants were included was not stated. Units which only rarely offered treatment were excluded.	Travel time	Odds of receipt of chemotherapy by travel time	Conditional logistic regression, adjusted for age, sex, tumour stage (where available), “site-specific characteristics” and deprivation. No tests for interaction or trend were performed. IMD deprivation score.
Laing ‘14 [45]	Scotland (Highland and Western Isles and Lothian)	Information Services Division and regional cancer datasets 2005 to 2010	3,308 men with prostate cancer who received treatment for prostate cancer, therefore Death Certified Only cases not included. No sites documented as excluded.	Rurality determined as Highland and Western isles resident compared with Urban (Lothian) residence. Treatment receipt compared by NHS health area.	Receipt of chemotherapy as ‘first treatment’ within the study period.	Student t-test, Mann–Whitney U test and two-sample Z test as appropriate. Stratified by risk group (e.g. low and intermediate compared with high-risk and metastatic). No deprivation indices.
McLeod, ‘99 [41]	Scotland	Hospital Discharge Data SMR01 linked to General Register Office death records. Jan ‘90 to June ‘94	15,016 colorectal cancer participants. Although not explicitly stated, it is probable that participants diagnosed histologically and clinically were included and cases diagnosed at post-mortem were excluded. Units which only rarely offered treatment were excluded.	Rurality of participants’ place of residence and hospital.	Odds of receipt of chemotherapy within 6 months of first admission by population density of patients’ residence (rural/urban) and by each hospital trust.	Multilevel logistic regression adjusting for age, sex, marital status, deprivation, type of admission, secondary diagnoses, hospital characteristics (e.g. chemotherapy availability) and severe illness. The final model was not clearly reported. Carstairs deprivation indices.
Monkhouse, ‘13 [32]	England	2010-2011	118 participants with upper GI cancer. Data from post-mortem necessarily excluded as patients recruited were from Multi-disciplinary meetings.	Hospital site, defined as ‘hub’ tertiary hospital or ‘spoke’ district general hospital	Time to receipt of chemotherapy from first multi-disciplinary meeting.	Parametric two-tailed t-test. No deprivation indices.
NLCA*’13 [28]	England, Wales, Scotland, N.Ireland and Jersey Hospital	NLCA ’12 data	40,216 lung cancer participants diagnosed histologically and excluding cases diagnosed at post-mortem. All cancer units included. Audit data includes clinically diagnosed cases, but not for outcomes reported here.	Cancer network and hospital trust	Numbers, percentages and Odds of receipt of chemotherapy in SCLC and Stage III/IV NSCLC PS 0/1 participants by hospital trust and cancer network.	Logistic regression, adjusted for age, sex, socioeconomic status, performance status and stage by cancer network or hospital trust. No deprivation indices.
Patel, ‘07 [38]	South East England	Thames Cancer Registry ‘94 to ‘03	67,312 participants diagnosed with lung cancer histologically or clinically and excluding cases diagnosed at post-mortem. All cancer units included.	Cancer Network.	Odds of receipt of chemotherapy within 6 months of first diagnosis by cancer network.	Logistic regression, adjusted for sex, age, type of lung cancer, cancer stage and deprivation. Tests for heterogeneity/trend were included as appropriate across categorical variables. IMD deprivation indices.
Paterson, ‘13 [42]	Southeast Scotland	Southeast Scotland Cancer Network colorectal database 2003-2009	4960 colorectal cancer patients. No mention of use of cases which are death certified only. No sites recorded as being excluded on base of size.	Health board of residence (in addition to individual characteristics such as deprivation)	Descriptive statistics as well as odds of receipt of chemotherapy.	Logistic regression, adjusted for age, sex, tumour site (colon or rectum), presence of metastatic disease at diagnosis, IMD score (Scotland) and health board.
Pitchforth, ‘02 [43]	Scotland	Scottish Cancer Registry ’92 to ‘96 linked to the Scottish Morbidity Record of inpatient and day cases (SMR01).	7,303 Colorectal cancer participants (histologically or clinically confirmed not specified). Cases diagnosed at post-mortem were excluded.	Rurality and distance to hospital of first admission.	Odds of receipt of chemotherapy within 6 months of first admission by hospital and by population density (rurality).	Multi-level regression, adjusted for age, sex, comorbidity, type of admission, death within first 6 months (as a marker of severity of illness) and deprivation. Participants were nested within areas, within hospitals. Distance was treated as an effect modifier. DepCat deprivation score.
			Units which only rarely offered treatment were excluded.			
Rich, ‘11 [39]	England	England NLCA and Hospital Episode Statistic (HES) data Jan ‘04-Dec 31 ‘08	7,845 Histologically confirmed SCLC participants. Units which only rarely offered treatment were also excluded It was not stated whether cases diagnosed at post-mortem were included.	Hospital trust	Odds of receipt of chemotherapy by hospital healthcare boundary	Multilevel logistic regression adjusted for age, sex, deprivation, performance status and stage and stratified by Charlson score of comorbidity. Townsend deprivation index.
Before and After Study						
Chamberlain ‘14 [30]	England and Wales	IMS Health data	Unknown number of individuals, data based on prescribing per head of population for England and Wales	Introduction of the Cancer Drugs Fund	Receipt of chemotherapy	Mg per 1000 population plotted using moving averages. Negative binomial regression. No deprivation score.
Stephens and Thomson, ‘12 [33]	England	IMS Health, England ‘09-‘11	Participants: All prescriptions of the five most commonly prescribed Cancer Drugs Fund drugs 2011. Likely included histological and clinically confirmed cases though not stated. Death certified only cases excluded. No units were excluded from analysis due to small numbers of cases.	Health authority	Mean volume, per head of population of prescribed cancer drugs fund chemotherapy in one year, by health authority. Variation expressed as 90th to 10th percentile differences.	Mean volumes dispensed for each drug (mg/ head population). Variation between SHAs: differences between the 10th and 90th percentile for each drug. No deprivation score.
Correlational Studies						
Richards, ‘04 [6]	England	IMS data for 16 NICE-approved cancer drugs, England NHS	IMS data for 16 NICE-approved cancer drugs. Death certified only cases were excluded. Likely included histological and clinically confirmed cases though not stated. No units were excluded from analysis due to small numbers.	Cancer network	Mean volume of prescribed chemotherapy by cancer network. Variation demonstrated by 90th to 10th percentile differences.	Mean prescribed volume (mg) per head of population per cancer network. Networks compared using 90th: 10th ratios. No deprivation score.

**Table 2:** Key Findings for the influence of time and distance to cancer treatment centre on chemotherapy access

Study	Un-adjusted OR (CI)	Adjusted OR for receipt of chemotherapy (CI)	*P*-value
Campbell ‘02 [29]	Medians and interquartile range shown. No unadjusted ORs presented.	Lung Cancer:	*P* value (global)
		≤5 km: 1.0	0.299
		6-37 km: 1.38 (0.61 to 3.14)	*P* value (trend)
		38-57 km: 1.93 (0.98 to 3.83)	0.166
		≥58 km: 1.43 (0.71 to 2.85)	
		Colorectal Cancer:	*P* value (global)
		≤5 km: 1.0	0.578
		6-37 km: 1.27 (0.66 to 2.45)	*P* value (trend)
		38-57 km: 0.91 (0.48 to 1.73)	0.517
		≥58 km: 1.37 (0.74 to 2.53)	
Crawford ‘12 [40]	Not shown	Rectal	Not stated
		Quartile 1: 1.0	
		Quartile 2: 0.702 (0.299 to 1.647)	
		Quartile 3: 0.858 (0.402 to 1.833)	
		Quartile 4: 1.058 (0.521 to 2.149)	
		Colonic	
		Quartile 1: 1.0	
		Quartile 2: 1.310 (0.730 to 2.352)	
		Quartile 3: 0.941 (0.540 to 1.639)	
		Quartile 4: 1.024 (0.617 to 1.697)	
		*adjusted for age and sex for stage 4 rectal cancer and colonic cancer	
Crawford ‘09 [36]	Not shown	Quartile 1: 1	-
		Quartile 2: 1.14 (0.96 to 1.34)	Not stated
		Quartile 3: 1.31 (1.11 to 1.55)	Q3 *P* < 0.01
		Quartile 4: 1.12 (0.95 to 1.32)	Not stated
Jones ’08 [31]	Not shown	Breast	
		Quartile 1: 1.0	-
		Quartile 2: 1.1 (0.95 to 1.2)	Not stated
		Quartile 3: 1.1 (1.0 to 1.2)	Q3 *P* < 0.05
		Quartile 4: 0.977 (0.89 to 1.1)	Not stated
		Colon	
		Quartile 1: 1.0	-
		Quartile 2: 1.1 (0.95 to 1.2)	Not stated
		Quartile 3: 0.89 (0.79 to 1.0)	Not stated
		Quartile 4: 0.882 (0.78 to 1.0)	Not stated
		Rectum	
		Quartile 1: 1.0	-
		Quartile 2: 1.1 (0.97 to 1.3)	Not stated
		Quartile 3: 1.1 (0.94 to 1.3)	Not stated
		Quartile 4: 0.828 (0.72 to 0.96)	Q4 *P* < 0.01
		Lung	
		Quartile 1: 1.0	-
		Quartile 2: 0.98 (0.88 to 1.1)	Not stated
		Quartile 3: 0.97 (0.88 to 1.1)	Not stated
		Quartile 4: 0.703 (0.63 to 0.79)	Q4 *P* < 0.01
		Ovary	
		Quartile 1: 1.0	-
		Quartile 2: 1.0 (0.86 to 1.2)	Not stated
		Quartile 3: 0.99 (0.84 to 1.2)	Not stated
		Quartile 4: 1.0 (0.88 to 1.2)	Not stated

**Table 3:** Key Findings for the influence of rural residence on chemotherapy access

Study	Un-adjusted OR (CI)	Adjusted OR for receipt of chemotherapy (CI)	*P*-value
Pitchforth ‘02 [43]	Numbers presented, but no unadjusted ORs presented	Rurality: adjusted OR 1.25 (0.99 to 1.51)	Not stated
		A post-hoc analysis of the effect of distance, grouped as <95 km and ≥95 km (straight line distance) and an interaction term for ‘no-cancer’ hospital. The results were “not statistically significant” although it is not shown.	
McLeod ‘99 [41]	Rurality: 0.77 (0.63 to 0.95)	Rurality: 0.88 (0.71 to 1.10)	Not stated
Laing ‘13 [45]	No OR presented, Read from figure: 3 % (rural) compared with 2 % (urban) *p* = 0.12.	Not adjusted	0.12

**Table 4:** Key Findings for the influence of country on treatment and access to chemotherapy

Chamber-lain ‘14 [30]	Chemotherapy prescribing volume ratios (PVR) compared for 15 drugs by country (England v Wales)	Not adjusted	*P* values pertain to receipt of one named chemotherapy in England compared with the same chemotherapy in Wales
	e.g. Bevacizumab PVR =3.28 (2.59 to 4.14) *P* < 0.001		
	For a full list of all 15 drugs please refer to the paper.

**Table 5:** Key Findings for the influence of designated Cancer Network of treatment and access to chemotherapy

Study	Un-adjusted OR (CI)	Adjusted OR for receipt of chemotherapy (CI)	*P*-value
Beckett ‘12 [34]	Not clearly stated	Not stated in text- read from figure: Range of network adjusted OR of receipt of chemo in SCLC 2.1 (CI 1.6 to 2.75) to 0.55 (0.49 to 0.75)	Not stated
Patel ‘07 [38]	Cancer network	Cancer network	*P* < 0.001
	A 18.0	A 18.0	
	B 18.2	B 20.5	
	C 18.6	C 20.7	
	D 17.4	D 18.6	
	E 18.6	E 20.5	
	F 24.1	F 27.7	
	G 17.4	G 16.6	
	H 10.8	H 10.3	
	I 10.4	I 10.9	
	J 18.0	J 16.9	
	K 7.8	K 6.1	
	L 15.0	L 12.6	
	M 14.3	M 14.2	
Richards ‘04 [6]	Variation by cancer network measured for each drug and adjusted by network size only. Values given per drug for variation across networks including 25 th/75 th percentile, 90 % ILE/10 % ILE, mean, median, maximum	90-percentile to/10-percentile volume ratios for drugs across cancer networks:	Not performed
		Rituxumab: 2.61, Imatinib 2.90, Gemcitabine 2.99, Fludarabine 3.15, Docetaxel 3.29, Capecitabine 3.60, Oxaliplatin 3.72, Irinotecan 3.73, Paclitaxel 3.78, Trastuzumab 4.25, Vinorelbine 8.13, Pegylated Liposomal Doxorubicin 9.69, Temozolamide 11.61, Cisplatin 2.26, Epirubicin 2.36, Doxorubicin 2.68	
NLCA ‘13* [28]	Numbers and percent of patients receiving chemo-therapy per network. Range: SCLC 49.3 % to 80.4 %, NSCLC 43.6 % to 70.9 %	Only one network was statistically significantly different to the whole NLCA population with SCLC: OR 1.88, 95 % CI 1.19 to 2.97. Nine cancer networks were statistically significantly different to the whole population odds for receipt of chemotherapy in NSCLC, with a range of 0.41 (0.27 to 0.60) to 1.93 (1.32 to 2.83) (in 2012).	Not stated

**Table 6:** Key Findings for the influence of designated Strategic Health Authority/Health Board of treatment and access to chemotherapy

Study	Un-adjusted OR (CI)	Adjusted OR for receipt of chemotherapy (CI)	*P*-value
Cartman ‘02 [35]	The proportion of patients having chemotherapy was 9.5 % (range 5 % to 12.9 %)	Adjusted analysis performed for survival regression analysis only	Not performed
Jack ‘03 [37]	Median % and range of chemo-therapy by health authority: chemo-therapy alone: 4 %, range 3-9 %, any chemo-therapy median 8 %, range 4-17 %.	Odds of chemotherapy if first hospital visited was a radiotherapy centre: OR 1.38 (1.06 to 1.80).	*P* = 0.018
Paterson ‘13 [42]	Unadjusted OR not given	Health board A, OR 1.00 (0.89, 1.38) (of any chemotherapy), Health board B OR 1.11 (0.89 to 1.38) *P* 0.36, Health board C OR 1.07, (0.91 to 1.25)	Health Board B 0.36
			Health Board C 0.42
Stephens ‘12 [33]	From figure.	Not performed	Not performed
	Everolimus 0.2 to 2.1 mg per head pre CDF across five SHAs reduced to 0.55 to 1.45 post-CDF; Lapatinib 0.05 to 2.0 pre-CDF and 0.70 to 1.25 after; Sorafenib 0.08 to 2.5 pre-CDF and 0.45 to 1.3 after; Bevacizumab and Cetuximab 0.25 to 2.1 pre and 0.45 to 2.0 post-CDF.

**Table 7:** Key Findings for the influence of designated Acute Trust of treatment and access to chemotherapy

Study	Un-adjusted OR (CI)	Adjusted OR for receipt of chemotherapy (CI)	*P*-value
Monkhouse ‘12 [32]	Not clearly presented. A table presents time to definitive oncology by cancer site for hib and spoke hospitals, however it is not entirely clear what the *P* value pertains to. The authors also note that “some different chemotherapy regimes take variable lengths of time so Table 4 is for illustrative purposes only”.	No evidence of adjustment	-
McLeod ‘99 [41]	Not quoted. Text states: “Without adjusting for patient, area and hospital level characteristics there was significant variation between both area of residence and hospital of first admission. Variation between hospitals was over six times that observed between areas.”	58 Scottish Hospital point estimates (with 95 % CI) for probability of receipt of chemotherapy presented in a figure.	Not stated
		OR of receipt chemotherapy by hospital (from figure): range ~ OR 0.55 to 7.5.	
NLCA* ‘13 [28]	Numbers and percent of patients receiving chemo-therapy per acute trust. Range (in trusts treating >10 SCLC cases): SCLC 20.8 % to 92.9 %, NSCLC 9.3 % to 45.9 %	Adjusted odds ratios for receipt of chemotherapy in SCLC by each acute trust (for trusts with >10 SCLC cases), compared with the whole NLCA population, showed a range of 0.24 (95 % CI 0.09 to 0.67) [18 cases] to 8.44 (95 % CI 1.79 to 39.80) [12 cases] with 14 trusts having statistically significant odds ratios demonstrating difference from the null- NLCA overall population estimate. Adjusted odds ratios for NSCLC by trust demonstrated a range of OR 0.20 (95 % CI 0.08-0.54) [25 cases] to 7.51 (95 % CI 1.69 to 33.4)[19 cases]. 27 trusts had statistically significant different odds of chemotherapy compared to the whole NLCA population for NSCLC.	Not stated
Rich ‘11 [39]	Not stated	Overall proportion receiving chemotherapy across NHS trusts was 0.61.	*P* < 0.001
		Range 0.14 to 0.86 (interquartile range 0.53 to 0.71). Adjusting for all patient features there was significant variation (*P* < 0.001)Trust odds ratios ranged from 0.03 (0.014 to 0.07) to 4.47 (1.46 to 13.72) with an interquartile range of 0.42 to 1.0.2	

### Characteristics of included studies

Twenty-four cohort studies [28–32, 34–43, 45], one correlational study [6] and two before and after studies [30, 33] were included in the review. Identified geographical exposure variables included: travel time or distance to a cancer treatment centre; rurality; and geographical area, defined by healthcare boundaries (e.g. acute hospital trust, cancer network or SHA). The majority of included studies relied on analysis of cancer registry data, with or without linkage to hospital records for co-morbidities [29, 31, 35–38, 40–42, 44]. Remaining data sources were IMS Health hospital prescribing data [6, 30, 33], local hospital data [32] and nationally collected audit data [34, 39, 46].

Populations of includedstudies varied. The majority of cancer registry studies used clinical or histologically confirmed cancers analysed in aggregate [31, 32, 36–38, 40] or separately [34, 35, 46]. One study restricted to histologically confirmed cancers only [39]. A further eight studies appeared to use both histological and clinically confirmed cancers together, but did not make this explicit [6, 29, 30, 33, 41–44].The majority of peer-reviewed studies excluded death certificated only (DCO) cancers [29, 31, 32, 35–39, 44]. Six peer-reviewed studies did not comment [30, 34, 40–43]. It is likely, based on study designs which relied on prescribing data in aggregate rather than individuals, that DCO cases were excluded in the non-peer reviewed literature [6, 33, 46]. Five studies excluded NHS hospital trusts and (their patients) from study where there were small numbers (<5 patients over study period [41, 43]; <30 patients [39]; <1 % of patients in the cohort [36]; or trusts with <1 % of treatment for each cancer type) [31]. Sample sizes ranged from 126 [32] to 117,097 participants, where stated [31].

Four different tools measuring deprivation were used in the included studies: Index of Multiple Deprivation (IMD) [28, 36, 38, 40, 42] Carstairs [29, 41], DepCat [43], Townsend [37, 39] or none [6, 32, 33, 45]. Rurality was defined and measured in different ways. Campbell et al. [29] defined rurality by the ‘distance to the nearest cancer centre in Aberdeen or Inverness, which, based on previous research implied low settlement size and rurality’. Laing described rural areas according to a “pre-existing classification” of an area as “accessible rural or remote-rural” (Highland and Western Isles) and compared it with Lothian, a “large urban or other urban” area. [45] Where these definitions of the rurality of the population are derived from is not included, though it is implied this may use the rural urban classification used by the UK Government [47]. McLeod [41] and Pitchforth et al. [43] defined ‘rurality’ using population density (persons per hectare) based on census data, which presumably coincides with Urban–rural classification, but has not been assessed.

Most studies presented adjusted odds ratios of receiving chemotherapy, based on multivariable logistic regression [29, 31, 34, 36, 37, 39–41, 43, 46] and/or crude proportions of receipt of chemotherapy (or timeliness of receipt of chemotherapy) [32] in eligible patients [6, 35, 38, 39, 45]. Twenty-one studies (including all NLCA) adjusted or stratified based on age, sex and deprivation [28, 29, 31, 34–43]. One time-trend analysis presented hazard ratios based on negative binomial regression and was not adjusted for age, sex, stage of participants [30]. Disease stage was absent from fiveof these studies [30, 35, 36, 41, 43], with one of the four using ‘disease extent’, which was not defined [35]. Performance status was only adjusted for in a minority of reports: all being based on NLCA data since 2009 [34, 39, 46], although attempts, such as using co-morbidity with linkage to Hospital Episode Statistics (HES) databases and admission type (elective or non-elective) were also used to approximate performance status in other studies [29, 41, 43].

### Reporting Clarity and Quality Assessment

The poorest reported areas were inclusion of a participant flow-chart; reporting of study design in the title or abstract; description of study result generalizability and missing data fields. Many papers did not report on efforts to limit bias. Additional file 3: Table S1.

‘NICE GATE adapted quality appraisals’ are presented in Additional file 4: Table S2–S8. Areas of potential selection bias, such as moving residence between cancer registration and treatment (cross-border flows) or the use of the ‘nearest’ geographical treatment centre to calculate distance, which may not be the treating hospital, was poorly considered in the majority of studies. Other potential selection biases included variability in the criteria for chemotherapy or poorly defining the population eligible for treatment in different geographical areas, inappropriately inflating or deflating some trusts/networks/SHA patient denominators. Finally, the exclusion of missing data may also introduce bias.

### Narrative Synthesis

Since meta-analysis was not possible due to the heterogeneity of included studies, a narrative synthesis relying more heavily on those studies with strong reporting and methodological quality follows.

### Distance travelled/travel time

Four studies examined distance or travel time and chemotherapy receipt (lung and colorectal cancer). No evidence of an association was demonstrated for distance to treatment centre and receipt of chemotherapy in a study based on low numbers (only 77 lung cancer patients and 114 colorectal cancer patients received chemotherapy). (15) Of three studies examining travel time to centre and receipt of chemotherapy, one study found no evidence of an association for colorectal cancer chemotherapy [40], but the remaining two studies had limited single quartile associations, which conflicted in the direction of their association [31, 36]. The largest study considered multiple cancer types [31] and found evidence of an association of *reduced* receipt of chemotherapy in the most distant category of time to treatment for two cancer types: Rectal (OR 0.8, 95 % CI 0.7 to 1.0 for the furthest distance quartile) and Lung cancer (0.7, 95 % CI 0.6 to 0.8, third most distant quartile). No test for trend was performed. The odds of receiving chemotherapy for small cell lung cancer were found to be *greater* for the third travel time quartile in a different study [36], OR 1.3, 95 % CI 1.1 to 1.6, with no other quartiles showing a statistically significant difference in receipt of chemotherapy and no test for trend.

### Rurality

There was no good evidence of an effect of ‘rurality’ on receipt of chemotherapy for colorectal cancer with three studies (two of which overlapped for two years of registry data) showing statistically non-significant trends of a positive association of increasing rurality with receipt of chemotherapy [41, 43]. Although one study appreared to show later cancer stage at presentation and poorer survival for rural popuations of men with prostate cancer, the disease stage was not adequately adjusted for and the “percentage of patients undergoing chemotherapy or watchful waiting” was the same same between the rural and urban populations (*p* = 0.12) [45]. However, the study did show a marginal difference in receipt of hormonal therapy between the rural Highlands and the Western Isles and the more urban Lothian (No adjustment by disease stage). The Highlands and Western Isles received less hormonal therapy, 16 % v 19 % respectively, *P* = 0.042 [45].

### Healthcare boundaries

#### Country

Only one study considered inter-country variation in cancer prescribing the UK. (Chamberlain et al.) [30] Prescribing of some high-cost cancer drugs was found to be up to seven times higher in England than in Wales. Only three of the fifteen drugs reviewed showed higher prescribing in Wales, compared with England- all three were drugs released around the time of the introduction of the Cancer Drugs Fund in England and were later found to be cost effective [30]. Results were not case-mix adjusted, but compared per 100,000 head of population only.

#### Strategic Health Authority (SHA, England) or Health Board (Scotland)

All four reports considering SHA-level variation found evidence of differences in cancer prescribing [33, 35, 37, 42].

Jack et al. [37] was the only included study which fully adjusted for case-mix (age at diagnosis, stage, histological sub-type, deprivation quartile and gender) and found rates of chemotherapy varied between 26 SHAs from 4 to 17 %. No test for statistical significance was performed for receipt of treatment by SHA.

#### Cancer Network

Cancer network level variation in chemotherapy prescribing was found in all studies in which it was examined [6, 28, 34, 38]. Only two studies quantified the inter-network variation with summary statistics, rather than a range. A chi-squared test for heterogeneity between cancer networks was performed (χ2 = 927.5, *P* < 0.001) in one study (8)where there was a more than four-fold difference between the lowest adjusted proportion receiving chemotherapy (6.1 %) and the highest (27.7 %). Variation was also expressed as a ratio of the 90th percentile to the 10th percentile of volumes prescribed in another: the findings suggested a range for inter-network variation from 2.6 fold variation (Rituximab) to an 11.6 fold variation (Temozolamide) [6]. Adjusted ORs for the most recent NLCA data, resulted in no evidence of a statistically significant different odds of receiving any chemotherapy in patients with Small Cell Lung Cancer (SCLC) in any cancer network, with the exception of one network (OR 1.9, 95 % CI 1.2 to 3.0). However, a much greater number of networks were statistically significantly different from the whole lung cancer audit population in the odds of receiving chemotherapy for Non-Small Cell Lung Cancer (NSCLC) (Stage IIIb/IV PS 0 or 1). Nine (of 30) cancer networks had statistical evidence to reject the null, with a range of 0.4 (0.3 to 0.6) to 1.9 (1.3 to 2.8) indicating nearly five times (4.7) greater prescribing in the highest, compared with the lowest prescribing cancer network [46].

#### Acute hospital trust

All four studies considering geographical variation at the Trust level also found evidence of variability in chemotherapy prescribing. There were, however, no summary values for the degree of heterogeneity or variation between the trusts in any of the reviewed studies. Two cohort studies [39, 41] assessed variability in access to chemotherapy for colorectal and lung cancer and found persistent variability, with the percentage of patients receiving chemotherapy per trust ranging from 0 to 68 %, with a median of 3 % in McLeod [41], compared with a range of 14 to 86 % (median of 62 %) in Rich et al. [39] for each cancer type. Adjusted odds ratios for receipt of chemotherapy in SCLC by each acute trust (for trusts with >10 SCLC cases), compared with the whole NLCA population, showed a range of 0.2 (95 % CI 0.1 to 0.7) to 8.4 (95 % CI 1.8 to 39.8) with 14 trusts having statistically significant odds ratios, rejecting the null of no difference between the trusts and the summary chemotherapy estimate for NLCA data [28]. 27 trusts showed statistically significant differences from the null for NSCLC (adjusted odds ratios demonstrated a range with OR 0.2 (95 % CI 0.1-0.5) to 7.5 (95 % CI 1.7 to 33.4)). Monkhouse et al. only compared two acute trusts performance, intending to represent a ‘hub’ and ‘spoke’ model of cancer care. When comparing receipt of chemotherapy alone it was not clear there was any statistically (or clinically) significant difference in receipt of treatment between the hub and spoke for oesophageal cancer, but there was uncertain evidence for a difference for gastric cancer (unclear data presentation) [32].

## Discussion

### Main findings

There is clear evidence of variation in chemotherapy use by geographical area, implying that even in the post NICE era, a postcode lottery in prescribing is still present. The cause of the geographical variation appears to be associated with healthcare boundaries, i.e. cancer network, and SHA level, associated with policy makers, commissioners and providers, rather than ‘natural geographic factors’ such as time or distance to chemotherapy centres, or rurality of the patient population. The best quality, case-mix adjusted studies demonstrate a 4–5 fold difference in chemotherapy receipt by network [38, 46].

### Strengths and Limitations (study and synthesis level)

Strengths of this review include the use of a systematic methodology to identify all relevant articles, including peer-reviewed, grey-literature and citation searching. There are no other systematic reviews pertaining to cancer drugs access in the NHS, despite the importance of this area for quality and equitable care in the NHS. Strengths at the study-level are the number of studies published in peer-reviewed sources which have attempted to adjust for differential case-mix.

The major limitation of this review was the challenge of designing a sufficiently inclusive search strategy in an area that has no pre-existing search filters. We were also unable to conduct a meta-analysis due to the heterogeneous study designs, which lack a unifying measure of geographical variation and variably adjusted for case-mix or stratified for deprivation. Arguably, by making the search strategy as inclusive as possible to capture all barriers to chemotherapy access, the search specific to geographical barriers did not use specific geographic key words and therefore may have missed some papers.

### Confounding

The most significant limitation of the included studies is the absence of case-mix adjustment, particularly stage and performance status. Those studies with no case-mix adjustment risk introduced bias where apparent differences (or lack of differences) between healthcare areas may refect differing health need in the study population. This bias may under-or over-estimate the relationship between geography and receipt of chemotherapy. Many studies adjusted for deprivation [29, 31, 37, 38, 40]; however, it is uncertain whether adjusting for this potential confounder may in fact artificially reduce and mask the association with geography and likelihood of receipt of chemotherapy.Stratifying by deprivation [36, 40] may have been a more appropriate way to accommodate the potentially modifying variable. In addition, studies used different deprivation indices (Carstairs, DepCat, IMD (or Scottish IMD) for adjustment. While these scales are similar, they have differing underlying assumptions, which may marginally contribute to differing findings. Finally, studies which are not based on population-based denominators, such as acute trusts, where the denominator reflects differential referral patterns, may result in non-equivalent populations which are challenging to compare. The review has therefore focused on cancer networks and health authority level data which have more comparable populations. The minor variations in study populations (e.g. uncertain use of DCO cases, restriction of included centres by size) do not clealy map to differences in outcome results. The differences clearly may contribute to a risk of bias and therefore studies are combined narratively, rather than statistically. Finally, although there are observed differences in chemotherapy utilisation, these differences cannot be translated into clinically ‘good’ or ‘bad’ outcomes. Outliers with high or low chemotherapy usage may both reflect poor clinical care [5, 6].

### Comparison with previous literature

Despite the number of papers describing variation nationally and internationally there have been no systematic reviews establishing the obstacles and facilitators to access to cancer drugs in the NHS or in any other publicly-funded health systems. One recent Canadian literature review [19] described 32 studies pertaining to inequitable access to cancer care (not solely pharmaceuticals) associated with geography including: variability between geographical regions affecting access to cancer treatments, rural vs urban barriers and distance/time travelled. Regional access to chemotherapy by healthcare boundaries for lung cancer was shown to vary between studies, supporting our review findings [19]. This review also described an association between greater distance to a cancer centre and reduced access to chemotherapy and palliative radiotherapy. Neither of the two papers referenced in the article provide evidence of an association between increasing distance and decreasing chemotherapy access, although there is an association with radiotherapy [48, 49]. The international literature also finds evidence of variability in chemotherapy access by healthcare boundaries, with less papers considering ‘natural’ factors of time or distance to treatment centre specifically [50].

The most comparable UK literature includes a recent review [2] which covers variability in access to NICE recommended pharmacotherapy, (not solely those specific to cancer). The literature review summarises some of the potential causes for the ‘gap’ between recommended and actual cancer prescribing in the UK, such as gaps in molecular testing by healthcare region (e.g. tests for the Epidermal Growth Factor Receptor [EGFR] mutation which is required before the prescription of some newer anti-cancer drug prescribing). Widely cited, grey literature UK publications prior to this review, which have been excluded due to inadequate outcome measures [51, 52], may have been misleading, having no case-mix adjustment and comparing geographical areas across different time periods, rendering the apparent three-fold (Scotland) and five-fold (Wales) reduction in access to chemotherapy compared with England hard to interpret. However, a peer-reviewed study, released in 2014 supported evidence of growing inter-country variation in chemotherapy prescribing between England and Wales following the introduction of the Cancer Drugs Fund (in England, not Wales) with an up to seven-fold difference in prescribing of chemotherapy [30].

### Implications for research and practice

International data have shown the UK to have a lower than average adoption of cancer drugs released in the last five years, when compared with other high-income countries [5]. In 2010 the UK government established the Cancer Drugs Fund to address this seeming ‘drugs shortfall’ in England. However, the devolved UK countries of Northern Ireland, Scotland and Wales did not introduce equivalent Cancer Drugs Funds. These different policy directions will logically increase the geographical variation between UK countries. The results of this review lend weight to the evidence that variation in chemotherapy prescribing is occurring at different healthcare boundaries, such as the variation in prescribing between England and Wales [30] and these may be more important policy areas to intervene for equitable prescribing than adjusting for time and distance travelled to cancer treatment centres. Distinguishing between explicit policy decisions which lead to differential prescribing and implicit conventions, such as local individual prescriber variation is important to ensure equity of access to chemotherapy. There are limited longitudinal analyses of variability in access to cancer drugs over time, which could improve understanding of the effects of various health reforms on utilisation of cancer drugs. The study of geographical variation would benefit from a standardised methodology and outcome measures for the conduct of studies, so that future studies may synthesise summary estimates of variation.

System process factors, including multi-disciplinary meetings, the role of assigned specialists, and cancer nurse specialists should be explored [53–56] as potential sources for variation in prescribing by geographical region. Finally, once variation in chemotherapy prescribing has been identified by geographical region, as in this review, the clinical significance of that variation, where both under- or over-use may be problematic, needs to be considered in the context of overall survival.

## Conclusion

Despite important heterogeneity in the included reviewed studies, there is consensus that there is variability in chemotherapy prescribing between geographical areas even after case-mix adjustment. Evidence that variation is greatest for NHS healthcare boundaries, rather than natural geographical entities, such as rurality, suggests that local treatment habits, capacity and policy may be more influential than natural geographical barriers to access.

## Additional files

Additional file 1:
**PRISMA 2009 Checklist.** (DOC 66 kb) Additional file 2:
**Medline Search strategy template** (DOCX 12 kb) Additional file 3:
**Table S1.** Quality of Reporting: STROBE checklist criteria. (DOCX 24 kb) Additional file 4:
**Table S2–S8**. NICE adaptaded GATEquality appraisal of included studies (DOCX 20 kb)
