# Supplementary material for: A systematic review of geographical variation in access to chemotherapy
Source: BMC Cancer. 2015 Dec 31;16:1. doi: 10.1186/s12885-015-2026-y (PMC4697930; doi:10.1186/s12885-015-2026-y)
Supplement: Additional file 1: — PRISMA 2009 Checklist. (DOC 66 kb) [file 12885_2015_2026_MOESM1_ESM.doc]

| **Section/topic** | **#** | **Checklist item** | **Reported on page #** |
| --- | --- | --- | --- |
| **TITLE** | | |  |
| Title | 1 | The title identifies the manuscript as a systematic review | 1 |
| **ABSTRACT** | | |  |
| Structured summary | 2 | A structured abstract is provided including: background; data sources; participants; study appraisal and synthesis methods; results and conclusions. | 2 |
| **INTRODUCTION** | | |  |
| Rationale | 3 | The rationale for the review is clearly stated in the Introduction section. | 3 |
| Objectives | 4 | An explicit statement of aims/questions being addressed is included as the final line of the introductory section. This aim is made more specific in the method section (study eligibility) where it refers to the included participants, study designs and outcomes. | 4, 5 |
| **METHODS** | | |  |
| Protocol and registration | 5 | Indicate if a review protocol exists, if and where it can be accessed (e.g., Web address), and, if available, provide registration information including registration number. | Not available |
| Eligibility criteria | 6 | Specify study characteristics (e.g., PICOS, length of follow-up) and report characteristics (e.g., years considered, language, publication status) used as criteria for eligibility, giving rationale. These items are all included under study eligibility and study selection. | 5, 6 |
| Information sources | 7 | Describe all information sources (e.g., databases with dates of coverage, contact with study authors to identify additional studies) in the search and date last searched. This is included in the search strategy page 5 | 5 |
| Search | 8 | Present full electronic search strategy for at least one database, including any limits used, such that it could be repeated. These items are all included under the search strategy and study eligibility sections. | 5 |
| Study selection | 9 | State the process for selecting studies (i.e., screening, eligibility, included in systematic review, and, if applicable, included in the meta-analysis). This is included in the study eligibility section page 5 | 5 |
| Data collection process | 10 | Describe method of data extraction from reports (e.g., piloted forms, independently, in duplicate) and any processes for obtaining and confirming data from investigators. This is included in the Data extraction section page 7 | 7 |
| Data items | 11 | List and define all variables for which data were sought (e.g., PICOS, funding sources) and any assumptions and simplifications made. This is included in the Data extraction section page 7. Due to word count all items were not included but as per Stroke checklist and table headings show abbreviated data variables. | 7 |
| Risk of bias in individual studies | 12 | Describe methods used for assessing risk of bias of individual studies (including specification of whether this was done at the study or outcome level), and how this information is to be used in any data synthesis. This is included in the Analysis section page 7 | 7 |
| Summary measures | 13 | State the principal summary measures (e.g., risk ratio, difference in means). This is included in the Data extraction section page 7. | 7 |
| Synthesis of results | 14 | Describe the methods of handling data and combining results of studies, if done, including measures of consistency (e.g., I^2^) for each meta-analysis. The Analysis sub-section under the methods and the Narrative synthesis section (p10) both describe the synthesis | 7, 10 |

Page 1 of 2

| **Section/topic** | **#** | **Checklist item** | **Reported on page #** |
| --- | --- | --- | --- |
| Risk of bias across studies | 15 | Specify any assessment of risk of bias that may affect the cumulative evidence (e.g., publication bias, selective reporting within studies). Risk of bias was formally assessed with the GATE NICE adapted appraisal tool. It is returned to in the discussion section. | 10, 15 |
| Additional analyses | 16 | Describe methods of additional analyses (e.g., sensitivity or subgroup analyses, meta-regression), if done, indicating which were pre-specified. | N/A |
| **RESULTS** | | |  |
| Study selection | 17 | Give numbers of studies screened, assessed for eligibility, and included in the review, with reasons for exclusions at each stage, ideally with a flow diagram. | Included as a separate attachment |
| Study characteristics | 18 | For each study, present characteristics for which data were extracted (e.g., study size, PICOS, follow-up period) and provide the citations. | Table 1 |
| Risk of bias within studies | 19 | Present data on risk of bias of each study and, if available, any outcome level assessment (see item 12). | Supplementary material |
| Results of individual studies | 20 | For all outcomes considered (benefits or harms), present, for each study: (a) simple summary data for each intervention group (b) effect estimates and confidence intervals, ideally with a forest plot. | Table 2 |
| Synthesis of results | 21 | Present results of each meta-analysis done, including confidence intervals and measures of consistency. This systematic review was not able to undertake a meta-analysis and included a narrative synthesis. | N/A |
| Risk of bias across studies | 22 | Present results of any assessment of risk of bias across studies (see Item 15). | Supplementary material |
| Additional analysis | 23 | Give results of additional analyses, if done (e.g., sensitivity or subgroup analyses, meta-regression [see Item 16]). | N/A |
| **DISCUSSION** | | |  |
| Summary of evidence | 24 | Summarize the main findings including the strength of evidence for each main outcome; consider their relevance to key groups (e.g., healthcare providers, users, and policy makers). Please see the discussion section: main findings, implications for research and practice sub-sections. | 14, 16, 17 |
| Limitations | 25 | Discuss limitations at study and outcome level (e.g., risk of bias), and at review-level (e.g., incomplete retrieval of identified research, reporting bias). In the discussion section under the strengths and limitations sub-section. | 14,15 |
| Conclusions | 26 | Provide a general interpretation of the results in the context of other evidence, and implications for future research. Please see discussion section comparison with pervious literature and implications for research and practice. | 15, 16, 17 |
| **FUNDING** | | |  |
| Funding | 27 | Describe sources of funding for the systematic review and other support (e.g., supply of data); role of funders for the systematic review. Funding listed under the acknowledgement section | 18 |

*From:*  Moher D, Liberati A, Tetzlaff J, Altman DG, The PRISMA Group (2009). Preferred Reporting Items for Systematic Reviews and Meta-Analyses: The PRISMA Statement. PLoS Med 6(6): e1000097. doi:10.1371/journal.pmed1000097

For more information, visit: **www.prisma-statement.org**.

Page 2 of 2
