# Supplementary material for: A systematic review of geographical variation in access to chemotherapy
Source: BMC Cancer. 2015 Dec 31;16:1. doi: 10.1186/s12885-015-2026-y (PMC4697930; doi:10.1186/s12885-015-2026-y)
Supplement: Additional file 2: — Medline Search strategy template (DOCX 12 kb) [file 12885_2015_2026_MOESM2_ESM.docx]

Search Strategy:

Database: **Medline** 1950 to present

--------------------------------------------------------------------------------

1 exp Antineoplastic Agents/ (260032)

2 exp Antineoplastic Protocols/ (96527)

3 Chemotherapy, Adjuvant/ (27083)

4 (cancer$ adj5 drug$1).tw. (16226)

5 (anticancer$ adj5 drug$1).tw. (14288)

6 antineoplastic.tw. (11531)

7 anti-neoplastic.tw. (1445)

8 chemotherap$.tw. (234338)

9 chemo-therap$.tw. (384)

10 exp Neoplasms/dt [Drug Therapy] (320357)

11 or/1-10 (572909)

12 health status disparities/ (5586)

13 Healthcare Disparities/ (5582)

14 exp Health Services Accessibility/ (77204)

15 exp Resource Allocation/ (14431)

16 exp "diffusion of innovation"/ (14112)

17 (diffusion adj5 (innovation$ or research)).tw. (739)

18 (diffusion adj5 (treatment$ or idea$)).tw. (518)

19 (diffusion adj5 product$).tw. (581)

20 or/12-19 (104200)

21 inequit$.tw. (3438)

22 inequalit$.tw. (11612)

23 (access adj5 treatment$).tw. (4337)

24 (access adj5 (therap$ or drug$)).tw. (4057)

25 disparit$.tw. (26698)

26 (access adj5 chemotherap$).tw. (221)

27 (equity or equities or equitable).tw. (8677)

28 (access adj5 (fair or unfair or fairness or unfairness)).tw. (126)

29 communication barriers/ (4378)

30 (barrier$ adj5 (treatment$ or care)).tw. (7246)

31 (ration or rationing or rationed).tw. (7616)

32 (equality or equalities).tw. (4033)

33 (allocat$ adj5 resource$).tw. (8810)

34 or/21-33 (84126)

35 20 or 34 (169767)

36 11 and 35 (1942)

Interrogated electronic databases included: MEDLINE, EMBASE, EconLit, Cinahl (Ebsco), PsychINFO (ovid), IBSS, Sociological abstract Social services Abstract (on proquest), AgeInfo and OpenSIGLE.

Informal approaches included: Google, a review of the Department of Health, (DoH) King’s Fund, NHS Confederation, UK NIHR Health Technology Assessment Programme, National Lung Cancer Audit, Rarer Cancers Foundation, MacMillan and Cancer Research UK websites.
