# Supplementary material for: A systematic review of geographical variation in access to chemotherapy
Source: BMC Cancer. 2015 Dec 31;16:1. doi: 10.1186/s12885-015-2026-y (PMC4697930; doi:10.1186/s12885-015-2026-y)
Supplement: Additional file 3: — Table S1. Quality of Reporting: STROBE checklist criteria. (DOCX 24 kb) [file 12885_2015_2026_MOESM3_ESM.docx]

| **Supplementary Table 1: Quality of Reporting: STROBE checklist criteria** | | | | | |
| --- | --- | --- | --- | --- | --- |
| STROBE statement heading | STROBE statement sub-heading | Studies meeting the criteria **(Total)** | Studies *incompletely* meeting the criteria **(Total)** | Studies where the criteria was omitted **(Total)** | Studies where the criteria was not relevant **(Total)** |
| **Title and Abstract** | *Study design (SD)* | Cartman ’02, **(1)** | Crawford ‘12, NLCA*, Beckett ‘12 **(11)** | Crawford ‘09, Jones ’08, McLeod ‘99, Patel ’07, Rich ‘11, Stephens ‘12, Jack ‘03, Campbell ‘02, Pitchforth ’02, Richards ‘04 Chamberlain ’14, Monkhouse ’12, Laing ’14, Paterson ’13 **(14)** | **(0)** |
|  | *Presence of Abstract* | Crawford ’12, Crawford ‘09, Jones ’08, McLeod ‘99, Patel ’07, Rich ‘11, Jack ‘03, Campbell ‘02, Cartman ’02, Pitchforth ’02, Chamberlain ’14, Laing ’14, Monkhouse ’12, Paterson ’13, NLCA* **(23)** | Richards ‘04, Beckett ‘12 **(2)** | Stephens, ’12, **(1)** | **(0)** |
| **Introduction** | *Background* | Crawford ’12, Crawford ‘09, Jones ’08, McLeod ‘99, Patel ’07, Rich ’11, Stephens, ’12,Jack ‘03, Campbell ‘02, Cartman ‘02, Pitchforth ’02, Richards ’04, Chamberlain ’14, Laing ’14, Monkhouse ’12, Paterson ’13, Beckett ’12 **(17)** | NLCA* (referred to website) **(9)** | **(0)** | **(0)** |
|  | *Objectives* | Crawford ’12, Crawford ‘09, Jones ’08, McLeod ‘99, Patel ’07, Rich ’11, Stephens, ’12,Jack ‘03, Campbell ‘02, Pitchforth ’02, Chamberlain ’14, Laing ’14, Monkhouse ’12, Paterson ’13, NLCA*, Richards ’04 **(24)** | Cartman ’02, **(1)** | Beckett ‘12 **(1)** | **(0)** |
| **Methods** | *Study design elements* | Crawford ‘09, Jones ’08, McLeod ‘99, Patel ’07, Rich ’11, Jack ‘03, Campbell ‘02, Cartman ‘02, Chamberlain ’14, Laing ’14, Paterson ’13, NLCA* **(20)** | Crawford ’12, Stephens, ’12, Pitchforth ’02, Richards ’04, Beckett ’12, Monkhouse ‘13 **(6)** | **(0)** | **(0)** |
|  | *Setting* | Crawford ’12, Crawford ‘09, Jones ’08, McLeod ‘99, Patel ’07, Rich ’11, Stephens, ’12,Jack ‘03, Campbell ‘02, Cartman ‘02, Pitchforth ’02, Richards ’04, Beckett ’12, Laing ’14, Monkhouse ’12, Paterson ’13, NLCA* (refers to website) **(25)** | Chamberlain ’14 **(1)** | **(0)** | **(0)** |
|  | *Participants* | Crawford ‘09, Jones ’08, McLeod ‘99, Patel ’07, Rich ’11, Cartman ‘02, NLCA*, Beckett ’12, Laing ’14, Monkhouse ’12, Paterson ’13, **(19)** | Crawford ’12, Stephens ’12, Jack ‘03, Campbell ‘02, Pitchforth ’02, Richards ’04, Chamberlain ’14, **(7)** | **(0)** | **(0)** |
|  | *Variables* | Crawford ’12, Crawford ‘09, Jones ’08, McLeod ‘99, Patel ’07, Rich ’11, Stephens ’12, Jack ‘03, Campbell ‘02, Cartman ‘02, Pitchforth ’02, Beckett ’12 Chamberlain ’14, Paterson ’13, NLCA* **(22)** | Stephens ’12, Richards ’04, Laing ’14, Monkhouse ’12, **(4)** |  | **(0)** |
|  | *Data sources/measurement* | Crawford ’12, Crawford ‘09, Jones ’08, McLeod ‘99, Patel ’07, Rich ’11, Stephens ’12, Jack ‘03, Campbell ‘02, Cartman ‘02, Pitchforth ’02, Richards ’04, Beckett ’12, Chamberlain ’14, Laing ’14, Monkhouse ’12, Paterson ’13, NLCA* **(26)** | **(0)** | **(0)** | **(0)** |
|  | *Bias* | Crawford ’12, Crawford ‘09, McLeod ‘99, Patel ’07, Rich ’11, Jack ‘03, Campbell ‘02, Beckett ’12, Chamberlain ’14 **(9)** | Jones ’08, Cartman ‘02, Pitchforth ’02, , NLCA*, Laing ’14, Monkhouse ’12, Paterson ’13, **(15)** | Stephens ’12, Richards ’04, **(2)** | **(0)** |
|  | *Study size* | **(0)** | **(0)** | **(0)** | Crawford ’12, Crawford ‘09, Jones ’08, McLeod ‘99, Patel ’07, Rich ’11, Stephens ’12, Jack ‘03, Campbell ‘02, Cartman ‘02, Pitchforth ’02, Richards ’04, Beckett ’12, NLCA ’13, Laing ’14, Monkhouse ’12, Paterson ’13, Chamberlain ‘14 **(26)** |
|  | *Quantitative variables* | McLeod ‘99, Patel ’07, Rich ’11, Stephens ’12, Jack ‘03, Cartman ‘02, Pitchforth ’02, Beckett ’12, Chamberlain ’14, Paterson ’13, **(10)** | Crawford ’12, Crawford ‘09, Jones ’08, Campbell ‘02, Laing ’14, Monkhouse ’12, NLCA* **(15)** | Richards ’04 **(1)** | **(0)** |
|  | *Statistical methods (a)* | Crawford ’12, Crawford ‘09, McLeod ‘99, Patel ’07, Rich ’11, Jack ‘03, Campbell ‘02, Beckett ’12 NLCA*, Richards ’04, **(18)** | Jones ’08, Stephens ’12, Cartman ‘02, Pitchforth ’02, Chamberlain ’14, Paterson ’13, Laing ’14, Monkhouse ’12, **(8)** |  | **(0)** |
|  | *Statistical methods (b) interactions and subgroups* | Crawford ’12, Crawford ‘09, Jones ’08, McLeod ‘99, Jack ‘03, Campbell ‘02, Cartman ’02, Beckett ’12,  NLCA* **(17)** | Stephens ’12, Pitchforth ’02, Chamberlain ’14, Paterson ’13, Laing ’14, Monkhouse ’12, **(6)** | Patel ’07, Rich ’11, Richards ’04 **(3)** | **(0)** |
|  | *Statistical methods (c) missing data* | McLeod ‘99, Patel ’07, Cartman ‘02, Pitchforth ’02, Beckett ’12, NLCA* **(14)** | Crawford ’12, Crawford ‘09, Jones ’08, Richards ’04, Chamberlain ’14, Paterson ’13, Laing ’14, Monkhouse ’12, **(8)** | Rich ’11, Stephens ’12, Jack ‘03, Campbell ‘02 **(4)** | **(0)** |
|  | *Statistical methods (d) loss to follow-up* | **(0**) | NLCA*, Beckett ’12 **(10)** | Stephens ’12, Richards ’04 **(2)** | Crawford ’12, Crawford ‘09, Jones ’08, McLeod ‘99, Patel ’07, Rich ’11, Jack ’03, Campbell ’02, Cartman ‘02,Pitchforth ’02, Chamberlain ’14, Paterson ’13, Laing ’14, Monkhouse ’12, **(14)** |
| **Results** | *Participants: no* | Crawford ’12, Crawford ‘09, Jones ’08, McLeod ‘99, Patel ’07, Rich ’11, Stephens ’12, Jack ‘03, Campbell ‘02, Cartman ‘02, Pitchforth ’02, Richards ’04, Beckett ’12, Paterson ’13, Laing ’14, Monkhouse ’12, NLCA* **(25)** | Chamberlain ’14, **(1)** | **(0)** | **(0)** |
|  | *Participants: reasons for non-participation* | n/a for retrospective studies Crawford ’12, Crawford ‘09, Jones ’08, McLeod ‘99, Patel ’07, Rich ’11, Jack ‘03, Campbell ‘02, Cartman ‘02, Pitchforth ’02, Paterson ’13, Laing ’14, Monkhouse ’12, **(22)** | NLCA*, Beckett ’12 **(2)** | Stephens ’12, Richards ’04 **(2)** | **(0)** |
|  | *Participants: flow chart* | **(0)** | **(0)** | Crawford ’12, Crawford ‘09, Jones ’08, McLeod ‘99, Patel ’07, Rich ’11, Stephens ’12, Jack ‘03, Campbell ‘02, Cartman ‘02, Pitchforth ’02, Richards ’04, Beckett ’12, NLCA*, Paterson ’13, Laing ’14, Monkhouse ’12, **(25)** | Chamberlain ’14, **(1)** |
|  | *Descriptive data: chrx* | Jones ’08, McLeod ‘99, Patel ’07, Rich ’11, Jack ‘03, Cartman ‘02, Beckett ’12, NLCA* Paterson ’13, Laing ’14, **(18)** | Crawford ’12, Crawford ‘09, Campbell ‘02, Pitchforth ’02, Chamberlain ’14, Monkhouse ’12 **(6)** | Stephens ’12, Richards ’04 **(2)** | **(0)** |
|  | *Descriptive data: no. missing participants by category* | McLeod ’99, NLCA*, Beckett ‘12 **(11)** | Crawford ’12, Rich ’11, Campbell ‘02, Pitchforth ’02, Richards ’04, Paterson ’13, Laing ’14, **(11)** | Crawford ‘09, Jones ’08, Patel ’07, Stephens ’12, Jack ‘03, Cartman ‘02, Chamberlain ’14, Monkhouse ’12, **(8)** | **(0)** |
|  | *Descriptive data: follow-up time summary* | Crawford ’12, Crawford ‘09, Jones ’08, McLeod ‘99, Patel ’07, Rich ’11, Stephens ’12, Jack ‘03, Campbell ‘02, Cartman ‘02, Pitchforth ’02, Richards ’04, Beckett ’12, NLCA*, Paterson ’13, Laing ’14, Chamberlain ’14, Monkhouse ’12, **(26)** | **(0)** | **(0)** | **(0)** |
|  | *Outcome data* | Crawford ’12, Crawford ‘09, Jones ’08, McLeod ‘99, Patel ’07, Rich ’11, Stephens ’12, Jack ‘03, Campbell ‘02, Cartman ‘02, Pitchforth ’02, Richards ’04, Beckett ’12, NLCA* Paterson ’13, Laing ’14, Chamberlain ’14, Monkhouse ’12, **(26)** |  |  | **(0)** |
|  | *Main results: unadjusted and adjusted est. Which confounders adjusted for and why* | McLeod ‘99, Patel ’07, Rich ’11, Jack ‘03, Campbell ‘02, Cartman ‘02, Pitchforth ’02, Beckett ’12, NLCA* **(14)** | Crawford ’12, Crawford ‘09, Jones ’08, Paterson ’13, Laing ’14, Chamberlain ’14, Monkhouse ’12, **(7)** | Stephens ’12, Richards ’04 **(2)** | **(0)** |
|  | *Category boundaries reported* | Crawford ‘09, Jones ’08, McLeod ‘99, Patel ’07, Rich ’11, Jack ‘03, Campbell ‘02, Cartman ‘02, Pitchforth ’02, Beckett ’12, NLCA* Paterson ’13, Chamberlain ’14, **(21)** | Crawford ’12, Laing ’14, Monkhouse ’12, **(3)** | Stephens ’12, **(1)** | Richards ‘04 **(1)** |
|  | *Translation of RR to AR (if relevant)* | **(0)** | **(0)** | Jack ’03, Cartman ’02, NLCA*, Richards ’04, Laing ’14, Monkhouse ’12 **(14)** | Crawford ’12, Crawford ‘09, Jones ’08, McLeod ‘99, Patel ’07, Rich ’11, Campbell ‘02, Cartman ‘02, Pitchforth ’02, Beckett ’12, Paterson ’13, Chamberlain ’14, **(12)** |
|  | *Other analyses* | McLeod ‘99, Patel ’07, Jack ‘03, Campbell ‘02, Pitchforth ’02, Richards ’04, Beckett ’12, NLCA* Laing ’14, Monkhouse ’12, Paterson ’13, Chamberlain ’14 (**20)** | Rich ’11, Stephens ’12, Cartman ’02 **(3)** | Crawford ’12, Crawford ‘09, Jones ’08, **(3)** | **(0)** |
| **Discussion** | *Results* | Crawford ’12, Crawford ‘09, Jones ’08, McLeod ‘99, Patel ’07, Rich ’11, Stephens ’12, Jack ‘03, Campbell ‘02, Cartman ‘02, Pitchforth ’02, Richards ’04, Beckett ’12, NLCA*, Laing ’14, Monkhouse ’12, Paterson ’13, Chamberlain ’14 **(26)** |  |  | **(0)** |
|  | *Limitations* | Crawford ’12, Crawford ‘09, Jones ’08, McLeod ‘99, Rich ’11, Jack ‘03, Campbell ‘02, NLCA*, Beckett ’12, Paterson ’13, Chamberlain ’14 **(19**) | Jones ’08, Stephens ’12, Cartman ’02, Pitchforth ’02, Richards ’04, Laing ’14, Monkhouse ’12, **(7)** | **(0)** | **(0)** |
|  | *Interpretation* | Crawford ’12, Crawford ‘09, Jones ’08, McLeod ‘99, Patel ’07, Rich ’11, Stephens ’12, Jack ‘03, Campbell ‘02, Richards ’04, Beckett ’12, Paterson ’13, Chamberlain ’14 **(13)** | Cartman ‘02, Pitchforth ’02, NLCA*, Laing ’14, Monkhouse ’12, **(13)** | **(0)** | **(0)** |
|  | *Generalisability* | Jones ’08, Rich ’11, Stephens ’12, Jack ‘03, Beckett ’12, NLCA*, Chamberlain ’14 **(15)** | McLeod ’99, Richards ’04, Laing ’14, **(3)** | Crawford ’12, Crawford ‘09, Patel ’07, Campbell ‘02, Cartman ’02, Pitchforth ’02, Monkhouse ’12, Paterson ’13, **(8)** | **(0)** |
| **Other Information** | *Funding* | Crawford ’12, Crawford ‘09, Jones ’08, McLeod ‘99, Patel ’07, Rich ’11, Stephens ’12, Jack ‘03, Campbell ‘02, Richards ’04, Beckett ’12, NLCA*, Chamberlain ’14, Laing ’14, Monkhouse ’12 (**23)** | Cartman ’02, (affiliations noted, not funding source) Pitchforth ’02, Paterson ’13, **(3)** | **(0)** | **(0)** |
