# Supplementary material for: A systematic review of geographical variation in access to chemotherapy
Source: BMC Cancer. 2015 Dec 31;16:1. doi: 10.1186/s12885-015-2026-y (PMC4697930; doi:10.1186/s12885-015-2026-y)
Supplement: Additional file 4: — Table S2–S8. NICE adaptaded GATEquality appraisal of included studies (DOCX 20 kb) [file 12885_2015_2026_MOESM4_ESM.docx]

| **Supplementary Table 2: NICE adapted GATE quality appraisal of included studies** | | | | | | | | | | | | | | |
| --- | --- | --- | --- | --- | --- | --- | --- | --- | --- | --- | --- | --- | --- | --- |
|  | **NLCA, ‘13** | **StephensP. & ThomsonD. ‘12** | **Crawford et al, ‘12** | **Beckett, P. et al, ‘12** | **Rich, AL. ‘11** | **Crawford et al, ‘09** | **Jones et al,**  **‘08** | **Patel, N. et al, ‘07** | **Richards M., ‘04** | **Jack, RH Et al, ‘03** | **Pitchforth E., ‘02** | **Campbell N.C., ‘02** | **CartmanML., ‘02** | **McLeod, A., ‘99** |
| Objective | Clear | Limited | Clear | Clear | Clear | Clear | Clear | Clear | Clear | Clear | Clear | Clear | Limited | Clear |
| Design | PC | B&A | RC | PC | RC | RC | RC | RC | Corr | RC | RC | RC | RC | RC |
| Population | ++ | NR | NR | ++ | ++ | NR | + | ++ | + | ++ | ++ | ++ | ++ | + |
| Represents source | ++ | + | + | ++ | ++ | + | ++ | ++ | + | ++ | + | + | ++ | + |
| Represents eligible population | ++ | + | + | ++ | ++ | ++ | + | ++ | + | ++ | + | ++ | ++ | + |
| Allocation of comparison | ++ | NA | ++ | ++ | + | ++ | ++ | ++ | + | ++ | ++ | ++ | ++ | ++ |
| Comparisondescribed | ++ | + | ++ | ++ | ++ | ++ | ++ | ++ | ++ | ++ | ++ | ++ | + | ++ |
| Concealed | NA | NA | NA | NA | NA | NA | NA | NA | NA | NA | NA | NA | NA | NA |
| Blinding | NA | NA | NA | NA | NA | NA | NA | NA | NA | NA | NA | NA | NA | NA |
| Exposure adequate | + | + | ++ | + | ++ | ++ | ++ | + | + | + | ++ | + | + | ++ |
| Contaminat-ion | + | + | ++ | + | ++ | ++ | ++ | + | + | + | + | + | + | + |
| Additional interventionconsidered | ++ | NR | + | ++ | ++ | + | + | ++ | + | ++ | + | NR | NR | NR |
| Lost to follow up | ++ | + | NA | ++ | NA | NA | NA | NA | + | NA | NA | NA | NA | NA |
| Represents setting | ++ | + | ++ | ++ | ++ | ++ | ++ | ++ | ++ | ++ | NA | ++ | ++ | ++ |
| Represents usual practice | ++ | ++ | ++ | ++ | ++ | ++ | ++ | ++ | ++ | ++ | ++ | ++ | ++ | ++ |
| Reliable outcomes | ++ | ++ | ++ | ++ | ++ | ++ | ++ | ++ | ++ | ++ | ++ | ++ | ++ | ++ |
| Outcomes complete | ++ | + | + | ++ | ++ | + | + | ++ | ++ | ++ | ++ | + | ++ | ++ |
| Important outcomes assessed | ++ | + | ++ | ++ | ++ | ++ | ++ | ++ | ++ | + | ++ | ++ | ++ | ++ |
| Outcomes relevant | ++ | ++ | ++ | ++ | ++ | ++ | ++ | ++ | ++ | ++ | ++ | ++ | ++ | ++ |
| Follow-up similar | ++ | ++ | ++ | ++ | ++ | ++ | ++ | ++ | ++ | + | ++ | ++ | ++ | ++ |
| Follow-up clinically meaningful | ++ | + | ++ | ++ | ++ | ++ | ++ | ++ | + | ++ | ++ | ++ | ++ | ++ |
| Baseline Groups similar | NR | - | + | NR | ++ | NR | + | + | NR | NR | NR | NR | NR | NR |
| ITT analysis | NA | NA | NA | NA | NA | NA | NA | NA | NA | NA | NA | NA | NA | NA |
| Sample size | NA | NA | NA | NA | NA | NA | NA | NA | NA | NA | NA | NA | NA | NA |
| Effect estimates | ++ | NR | ++ | NR | NA | ++ | ++ | ++ | + | ++ | NR | + | + | ++ |
| Appropriate analysis | ++ | - | + | NA | NA | + | - | + | - | ++ | + | + | - | + |
| Precise estimates | ++ | NR | ++ | NA | ++ | ++ | ++ | + | - | NA | + | + | - | + |
| Internally valid | ++ | + | ++ | ++ | ++ | ++ | + | + | + | ++ | + | - | + | + |
| Externally valid | ++ | + | + | ++ | ++ | + | + | + | + | ++ | + | - | - | + |

**Supplementary Table 2: NICE adapted GATE quality appraisal**

**Abbreviations**

**Corr**: Correlational Study

**PC**: Prospective Cohort

**RC**: Retrospective Cohort

**B&A**: Before and After Study

**++** “Indicates that for that particular aspect of study design, the study has been designed or conducted in such a way as to minimise the risk of bias.”(1)

**+** “Indicates that either the answer to the checklist question is not clear from the way the study is reported, or that the study may not have addressed all potential sources of bias for that particular aspect of study design.”(1)

**−** “Should be reserved for those aspects of the study design in which significant sources of bias may persist.”(1)

**NR** “Not reported: reserved for those aspects in which the study under review fails to report how they have (or might have) been considered”(1)

**NA** “Not applicable: reserved for those study design aspects that are not applicable given the study design under review (for example, allocation concealment would not be applicable for case control studies).”(1)

1. NICE. Methods for the development of NICE public health guidance (third edition) 2012. Available from: <http://www.nice.org.uk/article/pmg4/resources/non-guidance-methods-for-the-development-of-nice-public-health-guidance-third-edition-pdf>.

| **Supplementary Table 2: NICE adapted GATE quality appraisal of included studies following the updated electronic search** | | | | |
| --- | --- | --- | --- | --- |
|  | **Chamber-lain* ‘14** | **Monk-house ‘13** | **Pater-son ‘13** | **Laing ‘14** |
| Objective | Clear | Clear | Clear | Clear |
| Design | RC, B&A | RC | RC | RC |
| Population | + | ++ | ++ | ++ |
| Represents source | ++ | ++ | ++ | ++ |
| Represents eligible population | + | ++ | ++ | ++ |
| Allocation of comparison | ++ | + | ++ | + |
| Comparisondescribed | ++ | ++ | ++ | ++ |
| Concealed | NA | NA | NA | NA |
| Blinding | NA | NA | NA | NA |
| Exposure adequate | ++ | - | ++ | + |
| Contaminat-ion | + | + | + | + |
| Additional interventionconsidered | + | + | + | + |
| Lost to follow up | NA | + | NA | NA |
| Represents setting | ++ | + | ++ | ++ |
| Represents usual practice | ++ | ++ | ++ | ++ |
| Reliable outcomes | ++ | ++ | ++ | ++ |
| Outcomes complete | + | + | + | + |
| Important outcomes assessed | ++ | ++ | ++ | ++ |
| Outcomes relevant | ++ | ++ | ++ | ++ |
| Follow-up similar | ++ | ++ | ++ | ++ |
| Follow-up clinically meaningful | ++ | ++ | ++ | ++ |
| Baseline Groups similar | NR | ++ | NR | NR |
| ITT analysis | NA | NA | NA | NA |
| Sample size | NA | NA | NA | NA |
| Effect estimates | ++ | + | + | + |
| Appropriate analysis | ++ | + | + | + |
| Precise estimates | ++ | ++ | ++ | + |
| Internally valid | ++ | + | + | + |
| Externally valid | ++ | + | + | + |

*Note author’s own paper was included in the updated search strategy and therefore there is a conflict of interest in the quality appraisal of the paper. However, since all papers were included regardless of the quality score, this would not have biased the results.
